# Barriers to and facilitators for use of augmentative and alternative communication and voice restorative devices in the adult intensive care unit: a scoping review protocol

**DOI:** 10.1186/s13643-019-1232-0

**Published:** 2019-12-06

**Authors:** Laura Istanboulian, Louise Rose, Yana Yunusova, Franklin Gorospe, Craig Dale

**Affiliations:** 1 Lawrence S. Bloomberg Faculty of Nursing, University of Toronto, 155 College Street, Suite 130, Toronto, ON M5T 1P8 Canada; 2 Florence Nightingale Faculty of Nursing, Midwifery and Palliative Care, King’s College, 57 Waterloo Rd, Lambeth, London, SE1 8WA UK; 3 Department of Speech and Language Pathology, University of Toronto, 160-500 University Ave., Toronto, ON M5G 1 V7 Canada

**Keywords:** Communication, Critical care, Augmentative and alternative communication, Barriers and facilitators, Theoretical Domains Framework

## Abstract

**Background:**

Mechanically ventilated patients in the intensive care unit (ICU) experience profound communication impairment, placing them at risk for poor physical and psychological outcomes. Patient communication strategies such as augmentative and alternative communication (AAC) and voice restorative devices are recommended to facilitate communication. These strategies, however, are inconsistently adopted in ICU practice signaling utilization barriers. Our objective is to map and synthesize the current evidence-base for stakeholder-reported barriers and facilitators to patient communication strategy utilization for adults with an advanced airway in the ICU.

**Methods and analysis:**

We will use Arskey and O’Malley’s recommended methods to conduct a scoping review using a rapid review framework to streamline the process. A single reviewer will conduct a search and an initial screen of titles and abstracts from five electronic databases (MEDLINE, EMBASE, the Cochrane Library, Cumulative Index to Nursing and Allied Health Literature [CINAHL], and PsychInfo) from 1990 to present to identify English language peer-reviewed studies. Subsequently, two reviewers will independently screen a shorter list of studies for inclusion. We will also search the reference lists of eligible studies. Two reviewers will independently extract study characteristics, communication strategy, and stakeholder reported barriers and facilitators. We will code and categorize the extracted barriers and facilitators according to the Theoretical Domains Framework (TDF), an integrative framework of behavior change.

**Discussion:**

To our knowledge, this will be the first scoping review to map and synthesize reported barriers and facilitators to communication strategy utilization in the adult ICU using a theoretical framework. The results of this scoping review will help to identify trends and gaps in the current evidence-base and support recommendations for improving patient-centered practice, policy, and research related to successfully establishing ICU patient communication.

## Background

Treatment and monitoring of patients experiencing life-threatening conditions occur in the intensive care unit (ICU) [1]. The core treatment modality in the ICU is mechanical ventilation through an advanced airway interface, including an oral endotracheal or a tracheostomy tube. For patients requiring mechanical ventilation, the placement of an advanced airway interrupts vocalization and contributes to profound communication impairment. The shifting treatment paradigm from full to light or no-sedation has made communication impairment an increasingly important patient safety priority since less sedation results in patients who are more awake and struggling to be understood [2–5]. One study of mechanically ventilated patients across six specialty ICUs in an American academic health system estimated that over 50% of ICU patients have sufficient alertness to communicate with the people around them but may not be understood due to communication impairment arising from an artificial airway [6].

Mechanically ventilated patients describe communication impairment as one of the most stressful, dehumanizing, and frustrating events of hospital admission [7–9]. Communication impairment places ICU patients at risk for physical and psychological harm as it negatively impacts communication of disease symptoms, treatment responses, as well as participation in decision-making [6, 8, 10, 11]. Impaired patient communication is a modifiable risk factor for over- and under-recognition and treatment of symptoms of critical illness such as pain, anxiety, agitation, and dyspnea [6, 12]. Poor symptom management contributes to delirium, physical restraint use, and prolonged mechanical ventilation [13, 14]. In the long term, patient communication impairment also contributes to the development of chronic pain, depression, and post-traumatic stress disorder [10].

Patient communication in the ICU can be supported by strategies including augmentative and alternative communication (AAC) and voice restorative devices. AAC refers to all forms of communication, other than oral speech, that are used to express messages [15]. AAC can include “unaided” strategies (e.g., facial expressions, mouthing words, and gesturing) or “aided” strategies which include low-tech (e.g., alphabet or picture boards and writing instruments such as paper and pen) and high-tech (e.g., specialized computer communication interfaces) devices [15]. Phonation or voice restorative devices for patients with an advanced airway refer to voice enabling tracheostomy-based communication aids such as those that require cuff deflation (e.g., one-way speaking valves and ventilator-adjusted leak speech) and those that do not require cuff deflation (e.g., talking tracheostomy with dynamic cuff and fenestrated inner cannula) [16, 17]. Two recent systematic reviews report aided AAC strategies are effective in improving ICU patient satisfaction and reducing communication difficulty [18, 19]. Voice restorative devices are also demonstrated to enhance communication by restoring voice in the presence of an advanced airway [17].

Despite the range of available strategies and reported efficacy in reducing communication difficulties, patient communication strategies are inconsistently adopted in ICU practice [20–22]. Variable adoption of communication strategies in ICU practice signals the presence of utilization barriers [20–23]. A better understanding of the barriers to and facilitators for the utilization of patient communication strategies from the perspective of key stakeholders (e.g., patients, patient communication partners such as nurses, physicians, interdisciplinary clinicians, family members) can potentially improve patient communication strategy utilization in the adult ICU.

One recent systematic review reporting barriers to AAC use in the ICU only included experimental, quasi-experimental, and observational studies [18]. This review reported barriers to usage were device characteristics, the clinical condition of the patient, and constraints in staff time. The lack of inclusion of qualitative and mixed method approaches may inhibit understanding of key stakeholder perspectives and contextual issues relevant to the utilization of patient communication strategies in the ICU. Furthermore, previous reviews exploring barriers to patient communication in the adult ICU have not applied a theoretical framework to understand communication strategy utilization barriers and facilitators. The use of an implementation theory that includes behavioral and contextual determinants, such as the Theoretical Domains Framework (TDF), may help to better understand and identify potentially modifiable barriers to and facilitators for communication strategy utilization in the ICU [24, 25].

## Methods and design

### Aim and objectives

The primary aim of this scoping review is to answer the question: What are stakeholder reported barriers to and facilitators for patient communication strategy utilization for adults with an advanced airway in the ICU? To answer this question, we will conduct a scoping review with the primary aim of mapping of the current evidence on barriers to and facilitators for patient communication strategy (including AAC and voice restorative device) utilization for adults with an advanced airway in the ICU. The secondary aim of this review is to use the TDF to better understand barriers to and facilitators for communication strategy utilization, highlight key trends and gaps, and to inform evidence-based patient-centered practice, policy, and research. To our knowledge, this will be the first scoping review to map and synthesize reported barriers to and facilitators for to communication strategy utilization in the adult ICU using a theoretical framework.

## Methods

Our scoping framework will be informed by the scoping review methods suggested by Arskey and O’Malley, and advanced by Levac and Colquhoun [26–28]. We will follow the scoping review reporting methods outlined by the Preferred Reporting for Items for Systematic reviews and Meta-Analysis Protocols (PRISMA-P) (Additional file 1) [29]. We will also use rapid review methods to expedite the review process. A rapid review is a type of knowledge synthesis in which components of the systematic review process are simplified or omitted to produce information in a shorter period of time [30]. The rapid review components we will use include omission of gray literature and a single-reviewer system to perform the first screen of all titles and abstracts.

### Information sources

The research team developed a comprehensive search strategy (Additional file 2) in consultation with a health sciences information specialist. Our search strategy will be used in Ovid Medline Epub Ahead of Print, In-Process and Other Non-Indexed Citations, Ovid Medline Daily and Ovid Medline, Ovid Embase, and translated to Ovid PsycINFO, EBSCO Cumulative Index to Nursing and Allied Health Literature (CINAHL), and the Cochrane Library.

Including a complete gray literature search can be time consuming with very minimal relevant results and questionable replicability [31]. Omitting the gray literature as part of a rapid evidence assessment introduces the risk of selection bias [30, 31]. To mitigate the risk of missing relevant studies, we will hand search reference lists of included studies and recent systematic reviews and include those meeting inclusion criteria [18, 19, 30].

### Eligibility criteria

We will combine and deduplicate all electronic database searches in Endnote™ X9 [32]. One reviewer (LI) will perform an initial screen of titles and abstracts removing studies not meeting the eligibility criteria. The eligibility criteria are listed in Table 1. The remaining studies will be imported into Covidence™. Two independent reviewers (LI/FG) will screen study titles and abstracts against the eligibility criteria and subsequently screen full-text articles to aid in decision making about inclusion. Discrepancies will be resolved by discussion and a third reviewer (CD) as arbiter if necessary. Reference lists of included studies and recent systematic reviews will be screened by title and then full text by the process described above to decide about inclusion [18, 19].

**Table 1:** Inclusion and exclusion criteria

	Inclusion	Exclusion
Population/setting	• Adult (age 18+) ICU patients and their communication partners (i.e., nurses, physicians, interdisciplinary clinicians, family members) • ICU, specialized weaning centers, and high-dependency in patient settings • Patients with an advanced airway (oral endotracheal tube, tracheostomy)	• Emergency department, postoperative recovery units, hospital floors/wards, psychiatry, long-term care, and home settings
Intervention	• Patient communication strategies including AAC (unaided strategies, aided strategies (low- and high- tech); voice restorative devices	
Comparator	• Studies with comparison or no-comparison group will be included	
Outcomes	• Stakeholder (patients and communication partners including nurses, physicians, interdisciplinary clinicians, family members)—reported barriers and facilitators to patient communication strategies	• Studies without stakeholder reported barriers or facilitators to patient communication strategies
Type of study	• All study designs	• Editorials, letters, protocols, reviews, education pieces, reports, working papers, government documents, white papers, and evaluations

*AAC*, augmentative and alternative communication

We will include published studies from 1990 to present reporting quantitative, qualitative, mixed or multi-method designs, including both comparative (e.g., randomized, controlled, cohort, quasi-experimental) and non-comparative (e.g., survey, narrative, audit) methods. We will include all study designs to keep our search broad enough to capture diverse stakeholder reported barriers or facilitators of patient communication strategy utilization in the ICU. We will, however, exclude reviews, protocols, and opinion pieces including editorials and letters since these designs will likely not report our outcome of interest. For practical reasons, our search will be limited to English language studies. The 1990-year limit aligns with the paradigm shift to minimal ICU sedation practices. We also consider studies published more than 25 years ago may not be relevant to current barriers and facilitators to patient communication strategy utilization in the ICU.

### Data extraction

Two reviewers (LI/FG) will independently extract data using an iteratively developed data extraction form. Study identifiers and data to be extracted are listed in Table 2.

**Table 2:** Study identifiers and data to be extracted

• First author and year of publication • Full reference • Location of publication • Study design • Participant (e.g., patient, nurse, physician, interdisciplinary clinician, family) and unit characteristics • Stated aim(s) of study • Communication strategy (e.g., AAC unaided or aided, low or high tech, voice restorative device) • Verbatim description of barriers and facilitators to communication strategy utilization with reporting stakeholder noted • Stakeholder reported outcomes related to communication strategy utilization (e.g., ease of tool use; acceptability of tool; satisfaction; speech intelligibility)	

*AAC*, augmentative and alternative communication

Stakeholder-reported outcomes will be extracted if they are reported as barriers or facilitators to patient communication strategy utilization. *Stakeholders* include patients and their communication partners (e.g., nurses, physicians, interdisciplinary clinicians, family members). We will define *barriers* as any physiological, psychological, cognitive, or contextual conditions reported to reduce or negatively affect patient communication strategy utilization in the adult ICU [33]. We will define *facilitators* as physiological, psychological, cognitive, or contextual conditions reported to enhance or positively affect patient communication strategy utilization in the adult ICU [33].

We anticipate the extraction of the data will be an iterative process that depends on the evidence found in our search [27]. We also anticipate that data extracted will vary based on the type of study and data presented [27]. For example, for qualitative studies, descriptions of individual barriers and facilitators will be extracted verbatim unless only reported in a synthesized format. For quantitative studies, reported outcomes will be extracted and categorized as barriers or facilitators according to the approach described by Weatherson et al. (2017) (i.e., if 50% or more participants identify ease of use of a communication device/strategy, “ease of use” will be categorized as a facilitator) [34]. We will note the reporting stakeholder source of each extracted barrier and facilitator.

To ensure reliability, two reviewers (LI/FG) will pilot the barrier and facilitator extraction process then meet to compare findings. They will repeat this process for 3–5 articles, or until reliability is reached and the extraction tool is adapted. Upon completing the extraction, the two reviewers will meet to determine agreement on the presence or absence of barriers and facilitators within each paper. The authors will solve discrepancies through discussion, rereading source material and collaboration. In the event that agreement cannot be reached, the opinion of a third reviewer (CD) will determine the final result.

### Presentation of findings

Following the PRIMSA-ScR scoping review extension guide, we will present study screening and inclusion in a PRISMA chart [35]. We will also present a summary table of the studies meeting eligibility criteria including the stated aims of the study, study design, study setting, participant characteristics, and patient communication strategy. We will present extracted and coded barriers and facilitators both quantitatively and qualitatively.

### Synthesis of barriers and facilitators

Barriers and facilitators will be coded and categorized into the 14 domains of the TDF using a recommended coding approach [25, 36, 37]. A coding book will be developed by the research team prior to the coding process and iteratively modified to ensure accuracy, consistency, and transparency of the interpretive process of categorizing barriers and facilitators to the TDF domains [37]. Two reviewers will independently and deductively code the barriers and facilitators and will meet to discuss coding discrepancies until a final TDF categorization is accomplished. The resulting TDF categorization will be reviewed by the entire authorship team to determine if any alternative categorizations are plausible. We will then review the full list of extracted barriers and facilitators categorized each domain and determine the number of unique barriers and facilitators in each. Three criteria will be used to judge relevance of a TDF domain: (1) relatively high frequency of barriers or facilitators, (2) presence of conflicting barriers or facilitators, and (3) evidence of stakeholder beliefs that impact utilization of a communication strategy [25]. The authors will solve discrepancies through discussion, rereading source material, and collaboration.

We will present a quantitative summary of the barriers and facilitators to patient communication strategy utilization in the adult ICU including the frequency of reported barriers and facilitators in included studies (as counts within each TDF domain and proportions overall). We will also present the frequency (proportion) of barriers and facilitators in each TDF domain according to study design, communication strategy, and the stakeholder reporting the barrier or facilitator.

A qualitative analysis will be performed to provide major themes of barriers and facilitators of each TDF domain with reference to stakeholder and communication strategy [38]. Following an immersive reading of the coded TDF domains, we will look for recurring patterns in the data. Peer debriefing and reflexive writing will be used to bring meaning and coherence to barrier and facilitator concepts linking substantial portions of the data together. The overarching themes will represent patterns identified in the data comprising domain concepts most likely to influence communication behaviors including those that are potentially modifiable. Verbatim exemplars will be provided for the included TDF domains.

### Quality assessment

We will appraise the risk of bias of included studies using the Mixed Methods Appraisal Tool (MMAT)—Version 2018 [39, 40]. The MMAT is an easy to use tool demonstrating moderate to perfect interrater reliability [41]. Two reviewers (LI/FG) will independently appraise study quality. Though we will not exclude studies of low quality, we will use the results to describe the rigor of the included studies.

## Discussion

The primary aim of this scoping review is to answer the question: What are stakeholder reported barriers to and facilitators for patient communication strategy utilization for adults with an advanced airway in the ICU? To answer this question, we will map and synthesize stakeholder reported barriers and facilitators in the current peer-reviewed evidence-base to the domains of the TDF. We will build upon existing reviews by incorporating the qualitative and mixed methods literature, which may offer new stakeholder perspectives and contextual understandings. Since the TDF is a broad framework about behavior change that includes individual and environmental/contextual domains, it is a suitable framework to categorize the potential wide range of stakeholder reported barriers to and facilitators of communication strategy utilization in the adult ICU.

As physical and psychological harm can result from communication impairment in adult ICU patients treated with an advanced airway, it is critical to address this complex issue. The synthesis of barriers and facilitators using the TDF will allow us to better understand and highlight potentially modifiable antecedents to behavior change related to communication strategy utilization in the adult ICU. Furthermore, using the TDF to categorize barriers and facilitators reported in the current evidence base provides a theoretical foundation for future interventions targeting behavior change. The results of this review will also help to identify trends and gaps in the current peer-reviewed evidence base and support recommendations for improving patient-centered practice and policy related to successfully establishing and sustaining adult patient communication in the ICU.

### Strengths and limitations

The strengths of this scoping protocol include the use of a transparent and established scoping review methodology and reporting structure; a systematic search of five electronic databases developed in consultation with a health sciences information specialist; systematic screening and data extraction carried out by two independent reviewers for all steps except the initial screen; the inclusion of qualitative and mixed methods studies; the inclusion of a quality assessment step using the MMAT; and the use of a theoretical framework to map and synthesize barriers and facilitators to patient communication strategy utilization that spans multiple levels of influence in the adult ICU.

Limitations of our protocol include selection bias by restricting our search to publications in English after 1990, the use of a rapid review approach including omission of gray literature, and single reviewer first screen of titles and abstracts. To mitigate risk of selection bias introduced by our rapid evidence selection processes, we will follow recommended strategies to ensure replicability (methodological transparency), objectivity and accuracy (two independent screeners for second review of titles/abstracts and full text, two independent data extractors with a detailed process of reaching agreement, use of a quality assessment tool), and comprehensiveness (multiple databases and hand-searching of reference lists) [42, 43].

## Supplementary information

**Additional file 1.** PRISMA-P 2015 Checklist. Description of data: PRISMA-P Checklist.

**Additional file 2.** Results of the pilot database search. Description of data: Results of search performed March 17, 2019 in Ovid MEDLINE: Epub Ahead of Print, In-Process & Other Non-Indexed Citations, Ovid MEDLINE® Daily and Ovid MEDLINE® <1946-Present.

## Data Availability

All data generated or analyzed during this study will be included in the published scoping review article.

## Abbreviations

AAC: Augmentative and alternative communication
ICU: Intensive care unit
MMAT: Mixed Methods Appraisal Tool
TDF: Theoretical domains framework
