# Supplementary material for: Barriers to and facilitators for use of augmentative and alternative communication and voice restorative devices in the adult intensive care unit: a scoping review protocol
Source: Syst Rev. 2019 Dec 6;8:311. doi: 10.1186/s13643-019-1232-0 (PMC6896663; doi:10.1186/s13643-019-1232-0)
Supplement: Supplementary file 2 — Additional file 2. Results of the pilot database search. Description of data: Results of search performed March 17, 2019 in Ovid MEDLINE: Epub Ahead of Print, In-Process & Other Non-Indexed Citations, Ovid MEDLINE® Daily and Ovid MEDLINE® <1946-Present. [file 13643_2019_1232_MOESM2_ESM.docx]

Additional File 2: Results of the pilot database search. Performed March17, 2019 in Ovid MEDLINE: Epub Ahead of Print, In-Process & Other Non-Indexed Citations, Ovid MEDLINE® Daily and Ovid MEDLINE® <1946-Present>.

| Keyword and Subject Headings | Number of Publications Retrieved |
| --- | --- |
| \| 1 \| respiration, artificial/ or ventilator weaning/ \| \| --- \| --- \| \| 2 \| Ventilators, Mechanical/ \| \| 3 \| ((artificial* or mechanical*) adj2 (respirat* or ventilat*)).tw,kf. \| \| 4 \| ((assist* or support* or wean*) adj2 (respirat* or ventilat*)).tw,kf. \| \| 5 \| (invasive* adj2 ventilat*).tw,kf. \| \| 6 \| Tracheostomy/ \| \| 7 \| artificial airway.tw,kf. \| \| 8 \| Intubation, Intratracheal/ \| \| 9 \| (intubate* or extubate*).tw,kf. \| \| 10 \| trache*.tw,kf. \| \| 11 \| (endotrache* adj2 (tube or tubat* or ventilat*)).tw,kf. \| \| 12 \| (ventilat* adj3 (wean* or liberat*)).tw,kf. \| \| 13 \| (advance* adj2 airway*).tw,kf. \| \| 14 \| intra?tracheal.tw,kf. \| \| 15 \| intensive care units/ or coronary care units/ or respiratory care units/ \| \| 16 \| Intensive care unit*.tw,kf. \| \| 17 \| ICU.tw,kf. \| \| 18 \| respiratory care unit*.tw,kf. \| \| 19 \| long term acute care.tw,kw. \| \| 20 \| Critical Care/ \| \| 21 \| ((critical or intensive) adj3 nurs*).tw,kf. \| \| 22 \| Subacute Care/ \| \| 23 \| subacute care.tw,kf. \| \| 24 \| or/1-23 [concept 1: advanced airway and ICU] \| \| 25 \| Communication/ \| \| 26 \| communication barriers/ \| \| 27 \| communication.tw,kf. \| \| 28 \| (communicat* adj3 (capacity or readiness or ready or able or abilit* or impair* or efforts or effort or intent* or intervention*)).tw,kf. \| \| 29 \| (patient adj2 communicat*).tw,kf. \| \| 30 \| (communicat* adj3 disorders).tw,kf. \| \| 31 \| (Communicat* adj3 (capacity or Incapacity)).tw,kf. \| \| 32 \| (communicat* adj3 impair*).tw,kf. \| \| 33 \| (communicat* adj3 effort*).tw,kf. \| \| 34 \| (communicat* adj3 (able or abilit*)).tw,kf. \| \| 35 \| (verbal adj3 communicat*).tw,kf. \| \| 36 \| ((non?verbal or non?vocal) adj2 (communicat* or patient*)).tw,kf. \| \| 37 \| gestur*.tw,kf. \| \| 38 \| mouth*.tw,kf. \| \| 39 \| (lip adj2 read*).tw,kf. \| \| 40 \| voice*.tw,kf. \| \| 41 \| "rehabilitation of speech and language disorders"/ or language therapy/ or speech, alaryngeal/ or speech, esophageal/ or speech therapy/ or voice training/ \| \| 42 \| speech therap*.tw,kf. \| \| 43 \| ((alphabet or word or letter or picture or phrase) adj2 board).tw,kf. \| \| 44 \| ((augment* or assist*) adj2 (speech or vocal* or device* or communication or tool*)).tw,kf. \| \| 45 \| AAC.tw,kf. \| \| 46 \| Speech Production Measurement/ \| \| 47 \| ((communicat* or phonat* or speech) adj3 (assess* or apprais* or product* or evaluat* or measure*)).tw,kf. \| \| 48 \| (alternative adj3 communicat*).tw,kf. \| \| 49 \| (special* adj2 (talk* or trach*)).tw,kf. \| \| 50 \| ((speaking or speech*) adj3 (valve* or trach* or tube*)).tw,kf. \| \| 51 \| ((passey or one-way) adj2 valve*).tw,kf. \| \| 52 \| phonation/ or voice/ \| \| 53 \| phonat*.tw,kf. \| \| 54 \| (communicat* adj4 algorithm).tw,kf. \| \| 55 \| Communication Aids for Disabled/ \| \| 56 \| (communication* adj3 (aid or app* or board* or device* or tool*)).tw,kf. \| \| 57 \| electro?larynx*.tw,kf. \| \| 58 \| ((speech or speak*) adj3 (electronic* or synthesi*)).tw,kf. \| \| 59 \| ((fenestrat* or speech or speak*) adj2 trache*).tw,kf. \| \| 60 \| ((speech or speak*) adj2 valve).tw,kf. \| \| 61 \| Larynx, Artificial/ \| \| 62 \| (artificial adj2 larynx).tw,kf. \| \| 63 \| or/25-62 [communication impairment and devices/assessment] \| \| 64 \| 24 and 63 \| \| 65 \| Quality Improvement/ \| \| 66 \| quality improvement.tw,kf. \| \| 67 \| implement*.tw,kf. \| \| 68 \| ((Knowledge or evidence*) adj3 (translation or adopt*)).tw,kf. \| \| 69 \| Feasib*.tw,kf. \| \| 70 \| Barrier*.tw,kf. \| \| 71 \| Facilitator*.tw,kf. \| \| 72 \| intervention*.tw,kf. \| \| 73 \| or/65-72 \| \| 74 \| 64 and 73 \| \| 75 \| limit 74 to yr="1990 -Current" \| | 2750 |
